# The Effect of Omega-3 Docosahexaenoic Acid Supplementation on Gestational Length: Randomized Trial of Supplementation Compared to Nutrition Education for Increasing n-3 Intake from Foods

**DOI:** 10.1155/2015/123078

**Published:** 2015-08-27

**Authors:** Mary A. Harris, Melanie S. Reece, James A. McGregor, John W. Wilson, Shannon M. Burke, Marsha Wheeler, Jennifer E. Anderson, Garry W. Auld, Janice I. French, Kenneth G. D. Allen

**Affiliations:** ^1^Department of Food Science and Human Nutrition, Colorado State University, Fort Collins, CO 80523, USA; ^2^Department of Neonatology, University of Colorado Hospital, Denver, CO 80045, USA; ^3^Department of Obstetrics and Gynecology, Denver Health Medical Center, Denver, CO 80237, USA; ^4^Department of Obstetrics and Gynecology, University of Colorado Hospital, Denver, CO 80045, USA; ^5^LA Best Babies Network, Los Angeles, CA 90015, USA

## Abstract

*Objective*. DHA supplementation was compared to nutrition education to increase DHA consumption from fish and DHA fortified foods.* Design*. This two-part intervention included a randomized double-blind placebo controlled DHA supplementation arm and a nutrition education arm designed to increase intake of DHA from dietary sources by 300 mg per day.* Setting*. Denver Health Hospitals and Clinics, Denver, Colorado, USA.* Population*. 871 pregnant women aged 18–40 were recruited between16 and 20 weeks of gestation of whom 564 completed the study and complete delivery data was available in 505 women and infants.* Methods*. Subjects received either 300 or 600 mg DHA or olive oil placebo or nutrition education.* Main Outcome Variable*. Gestational length.* Results*. Gestational length was significantly increased by 4.0–4.5 days in women supplemented with 600 mg DHA per day or provided with nutrition education. Each 1% increase in RBC DHA at delivery was associated with a 1.6-day increase in gestational length. No significant effects on birth weight, birth length, or head circumference were demonstrated. The rate of early preterm birth (1.7%) in those supplemented with DHA (combined 300 and 600 mg/day) was significantly lower than in controls.* Conclusion*. Nutrition education or supplementation with DHA can be effective in increasing gestational length.

## 1. Introduction

Evidence from both human and animal studies suggests that essential fatty acids of the n-6 and n-3 series play important and modifiable roles in maintaining gestation. In early epidemiological studies [1, 2] erythrocyte (RBC) n-3 docosahexaenoic acid (DHA) correlated with gestational age. Previous studies in our laboratory have shown that women delivering prematurely had markedly decreased n-3 DHA and elevated n-6 arachidonic acid (ARA) and n-6 linoleic acid (LA) pools in both RBC membranes and plasma phospholipids and increased RBC membrane n-6 docosapentaenoic acid (DPA), a biomarker for n-3 fatty acid insufficiency [3]. In an early randomized supplementation trial, pregnant women supplemented with 2.7 g EPA + DHA/day throughout the second half of gestation experienced a significant 4.5-day increase in gestational length [4]. A food based supplementation trial in pregnant women demonstrated that 137 mg of DHA per day from 24–28 weeks until delivery increased gestational length by a significant 6 ± 2.3 days [5]. The FOTIP [6] trials of fish oil supplementation in high risk pregnancy demonstrated a reduction in the recurrence of preterm birth from 33% to 22%, OR .54 (95% CI, 0.30, 0.98), and resulted in an 8.5-day increase in gestational length and significant increases in infant birth weight. A recent report demonstrated that supplementation with 600 mg/day DHA from the 20th week of pregnancy resulted in a 2.9-day increase in gestational length accompanied by increased birth weight, length, and head circumference reduction in preterm birth at <34 weeks [7]. However, results from randomized controlled trials remain inconclusive [8]. Several meta-analyses failed to show any effects of DHA or fish oil supplements on gestational length and birth weight [9, 10]. Data from the maternal-fetal network trials of 17-hydroxy progesterone and fish oil for the prevention of preterm birth were unable to show an additive effect of high doses of fish oil on reduction in the recurrence of preterm birth [11]. However, an examination of fish intake in this group showed that intake of moderate amounts of seafood was associated with increased gestational length [12]. Overall, the effect of fish intake on reduction in preterm (PT) birth has been more consistent than the effect of fish oil or DHA supplements and questions about the effect of DHA supplementation in pregnancy outcomes remain unanswered. Therefore, specific objective of this study was to compare supplementation at two levels of DHA oil to nutrition education targeted to increase DHA consumption from fish and DHA fortified foods on gestational length.

## 2. Methods

The study was a two-part intervention which included a randomized double-blind placebo controlled supplementation arm in which subjects received either 300 or 600 mg of algal derived DHA or olive oil placebo and a nutrition education arm designed to increase intake of DHA by 300 mg per day from fish and other dietary sources. Approval was obtained from the Human Research Committee of Colorado State University and Colorado Multiple Institutional Review Board.

### 2.1. Subjects

The sample size was determined on the basis of the number of subjects needed to detect a 5-day difference in gestational age at the *p* < 0.05 level. 871 subjects were recruited from Denver Health Hospitals (Denver, CO) antenatal clinics. Four of the largest clinics were selected to participate in the study. Three clinics were randomized to receive supplements and one to receive nutrition education. For the supplemental arm, 662 subjects were recruited and of these 28 were ineligible, 634 subjects were randomly allocated to treatment, 289 withdrew, and complete delivery data was available on 345 subjects in the supplemental arm. 209 subjects were recruited and consented to participate in nutrition education. Birth data was available on 191 of these and 18 were lost to follow-up. In total, complete delivery data was available for 536 of the 563 subjects (Table 1). Baseline demographic and anthropometric characteristics of subjects (Table 2) in the four groups were not significantly different and there were no differences in between those who completed the study and those who withdrew. Patients were enrolled during regularly scheduled prenatal visits at 16 to 20 weeks of gestation or at WIC intake visits. Subjects with singleton pregnancies were eligible for the study if they were 18 years of age or older, willing to participate, and able to sign informed consent and HIPPA forms in English or Spanish. Subjects were excluded from the study if they presented with known medical or obstetrical complications associated with increased risk for preterm birth including cervical incompetence, presence of cervical cerclage, placenta previa, intrauterine infection, known substance abuse, multiple fetuses, current preeclampsia, preexisting diabetes, or a history of gestational diabetes in a prior pregnancy. Subjects were also excluded if they were taking nonsteroidal anti-inflammatory drugs (NSAIDS) or if they consumed salmon, mackerel, rainbow trout, or sardines at least once weekly or if they had known allergies to fish or any constituent of the nutritional supplement.

### 2.2. Supplemental Arm

Subjects were recruited by study professional research associates and allocated to one of three treatment groups (300 mg DHA, 600 mg DHA, or placebo) using a stratified block randomization schedule, generated using a randomization table by staff at Martek Biosciences, to insure equal group assignment from each of three clinics participating in the supplement trial. DHA was provided in the form of 300 Kcal supplement bars containing DHASCO-S oil. Gel capsules containing the test oil or olive oil were available for those who refused the bars. Bars and gel capsules were provided by Martek Biosciences, Columbia, MD. Supplementation was initiated at week 20 of gestation and continued until delivery. Both subjects and all study personnel were blinded as to treatment. DHA content of the bars and capsules was verified by gas chromatography.  Table 3 contains fatty acid analysis of the DHASCO-S oil. Compliance was determined from return of study bars or capsules at regularly scheduled intervals and after delivery at the first postnatal visit and averaged 76–81%.

Prepackaged supplements were stored in cool secured locations, accessible only to the investigative team. A four- to six-week supply of supplements was dispensed at each prenatal visit and subjects were instructed to return any unused supplements. Records of the supplements dispensed and returned were used to calculate adherence with treatment. In addition, each subject kept a supplement diary and research assistants reviewed supplement diaries at each prenatal visit and collected data on side effects, dietary intake of DHA-rich foods, and adverse events including vaginal infections, vaginal bleeding, and preterm labor occurring during pregnancy. Following delivery, maternal and infant medical records were reviewed for adverse antenatal and intrapartum events in the supplemental arm. The maternal record provided date of delivery, estimated delivery date, maternal height and weight at first prenatal visit, mode of delivery, type of rupture of membranes (ROM), type of labor onset, estimated blood loss, and complications of labor and delivery. The newborn record provided information on infant gender, birth weight, birth length, and head circumference. Gestational length was determined using the difference between the estimated delivery date and the actual delivery date. Gestational length was determined using the difference between the delivery date established using a combination of the LMP method and ultrasound, where available, and the actual delivery date.

### 2.3. Erythrocyte DHA Levels

Blood samples were obtained at enrollment between 16 and 20 weeks of gestation and at delivery. Seven mL blood samples were drawn in EDTA-containing vacutainer tubes. Samples were separated by centrifugation (1600 g, 10–15 min at 25°C) within four hours of delivery and flushed with nitrogen gas, frozen in liquid nitrogen, and stored at −80°C for later batched analysis of phospholipid fatty acids. Plasma lipids were extracted in chloroform/methanol (2 : 1 v : v). Fatty acid methyl esters (FAME) will be prepared by transmethylation with 14% boron trifluoride in methanol (Sigma Chemicals, St Louis, MO). FAME were analyzed by temperature-programmed gas liquid chromatography using a 30 m × 0.25 mmID microcapillary column and identified using reference standards (Nu-Check Prep, Elysian, MN).

**Figure 1:**
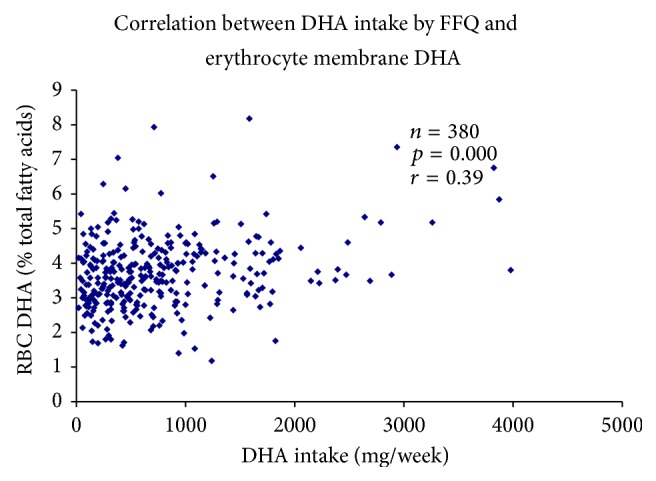
Validation of food frequency inventory with erythrocyte (RBC) membrane percent total DHA.

Dietary intake of long chain n-3 fatty acids was estimated at enrollment (16–20 weeks), using a pictorial food frequency inventory developed for this study. The food frequency was validated against RBC DHA in 340 subjects shown in Figure 1 (*r* = 0.39, *p* < 0.005). The inventory contained a finite list of foods which are the rich sources of long chain n-3 fatty acids (≥1.0 mg/100 g edible portion of DHA +/or EPA) and DHA-enriched functional foods and eggs. The food inventory was quantitated using the USDA Nutrient Data Base (release 15).

Nutrition Education Intervention: subjects were recruited from a fourth Denver Health, Women Infants and Children's (WIC) clinic, which was not participating in the clinical trial to avoid contamination of the supplemental groups by nutrition information. Convenience sampling was used to recruit subjects at WIC clinic visits between 18 and 20 weeks of gestation to participate in the nutrition education component of the study. Subjects received educational materials which were developed using focus group data collected in this population and based upon the Health Belief Model. This process and resulting materials are described elsewhere [13]. The education materials included a daily reminder/planner with DHA information, a refrigerator magnet, shopping lists, recipes, recipe holders, and personalized stickers for use in the daily planner. Materials also included a short (one-page) printed nutrition education flyer discussing the benefits of omega-3 fatty acids. Each month, participants received a mailing that included additional recipes and DHA-enriched egg coupons. A choice of several cans of DHA-rich fish (albacore tuna, sardines, and pink salmon) was given to each participant at the time of enrollment. Adherence to nutrition education was established by tracking egg coupon redemption over time and by follow-up telephone interviews of participants. An average of 11 dozen egg coupons per participant were redeemed which represented availability of 6 eggs per subject per week.

### 2.4. Statistical Analysis

The primary outcome variables tested were gestational length (in days). Secondary outcomes, infant birth weight, birth length and head circumference, percent preterm birth, and percent postterm birth were evaluated. Continuous variables were analyzed using analysis of covariance (ANCOVA) and regression analysis using the SAS statistical package version 14. An intent to treat analysis was conducted using SAS phreg and lifetest procedures. Covariates used in the ANCOVA, maternal prepregnancy BMI, and ethnicity were those shown to have significant effects on gestational days in the univariate analysis. Dichotomous variables were analyzed using the IBM SPSS Statistics 20, Pearson Chi-Square analysis.

## 3. Results

To examine the difference in effectiveness of DHA supplements in the form of gel capsules compared to the food bars, analysis of differences in gestational length by supplement type was conducted. Fifty-one subjects consumed capsules as a substitute for bars. There was no difference in response within each group attributable to supplement type (*p* = 0.773). The intent to treat analysis showed that there were no significant differences in ethnicity or other characteristics of those who completed the study compared to those who withdrew (Table 1). Ethnicity by group in those who completed and withdrew is shown in Table 3. The population was 78.5% Hispanic and did not vary between those who completed and withdrew from the study. However, because ethnicity varied among treatment groups and gestational length and birth weights of Hispanic babies were higher than all others, ethnicity was used as a control variable in the ANCOVA of gestational length and birth weight. Gestational length, birth weight, birth length, and head circumference are shown in Table 4. There was a significant (*p* = 0.025 for the model) 4-day increase in gestational length with 600 mg (*p* = 0.025 DHA supplement) and 4.5 days with nutrition education (*p* = 0.003) compared to controls (Table 4). There was a 4-day increase in gestational length with the 300 mg supplement which approached significance (*p* = 0.065). The differences between 600 mg DHA supplement and nutrition education groups were not significant.

Four infants were born postterm (>294 days), one in each of the supplement groups and two in the nutrition education group. Labor was induced at 41 weeks in thirteen subjects, four supplemented subjects (all in the 300 mg DHA group) compared to six in the placebo and three in the education group (Table 4). There were no significant increases in the total number of subjects who had induced labor at any gestational age nor any differences in parity among supplementation groups (Table 5). Fifty-one infants (9.5%) were born preterm (<280 days) and there were no significant differences among groups. Fourteen infants were born at less than 34 weeks of completed gestation, two in each of the DHA groups (1.7%), three in the nutrition education group (1.8%), and seven (5.7%) in the placebo group. These rates were not found to be significantly different by Chi-square analysis (*p* = 0.3). In order to test the effect of any amount of DHA in the controlled trial, 300 and 600 mg DHA treatment groups were combined. When supplement groups were combined, the preterm birth rate in those receiving any DHA (300 or 600 mg/day) was not significantly different from placebo. The early preterm birth rate of 1.7% in those supplemented with any DHA (300 or 600 mg/day) was significantly lower than the early preterm birth rate of 5.7% in the placebo group (*p* = 0.045 by two-tailed Chi-square analysis).

Mean birth weight, birth length, and head circumference with supplementation or nutrition education did not differ from the control or among groups. Maternal RBC DHA significantly increased in supplemented groups and decreased in controls. Maternal RBC % total DHA at delivery positively correlated with gestational length (*p* < 0.01  *r* = 0.15) and birth weight (*p* = 0.05  *r* = 0.11). Each 1% increase in RBC DHA was associated with a corresponding 1.6-day increase in gestational length. Maternal RBC % total arachidonic acid at delivery was not correlated with gestational length or birth weight.

Dietary DHA intake among Non-Hispanic Whites averaged 69 mg/day, African American 161 mg/day, Hispanics 143 mg/day, and others 119 mg/day. In those who completed the study, adherence to supplement ranged from 75% to 80% and did not significantly differ among groups.

RBC membrane DHA levels in the supplemented groups (expressed as percent total fatty acids) were not significantly different at entry (Table 1). Mean DHA levels (±SD) were significantly increased at delivery with either the 300 mg or 600 mg supplement (3.88 ± 1.61 and 4.19 ± 1.50, resp.) compared to CON (3.09 ± 1.19).

Given the broad inclusion criteria and the potential for pregnancy complications from altering maternal fatty acid consumption, we recorded and analyzed a large variety of adverse effects. The most serious of these included late miscarriage, stillbirth, and early neonatal demise and each of these is due to extreme immaturity at delivery. In each of these cases, delivery occurred after enrollment but before consumption of supplements. In each instance women removed themselves from the study and were thus included only in the “intent to treat” analysis. Other obstetrical outcomes included cervical insufficiency, primary intra-intrauterine infection, placenta previa necessitating expedited surgical delivery, and an undiagnosed uterine anomaly and were deemed not to be study related. There were only 3 cases of severe preeclampsia (1 in each group of the supplement arm) and a very low incidence of postpartum bleeding and no difference in blood loss among supplementation groups. Events at delivery (Table 6) including premature rupture of membranes (PROM), preterm premature rupture of membranes (PPROM), spontaneous rupture of membranes (SROM), and augmented rupture of membranes (AROM) and type of labor were analyzed in the supplement arm. Supplementation showed no significant negative effects of DHA supplements.

## 4. Discussion

The data are in agreement with several studies of fish oil supplementation at higher doses in which average 4- to 4.5-day increases in gestational length were observed [4–8]. It is probable that there a ceiling effect exists at about 600 mg DHA/day. The increase in gestational length in this study with 600 mg/day or nutrition education was lower than the 6-day increase demonstrated in a small study in which subjects were supplemented with 165 mg of a food source of DHA [5] but comparable to a study with 600 mg of the identical source of supplemental DHA, which reported a 2.9-day increase in a similar number of subjects [7]. This difference may be attributable to the relatively low risk for preterm delivery and low birth weight in the Hispanic population compared with a different ethnic composition of the low income population or the relatively small number of subjects in the former food based study. Alternatively, it is possible that food based DHA has a greater effect on gestational length, although that was not evident comparing our food based nutrition education group to supplements in this study.

To date, two systematic analyses did not show a statistically significant effect of n-3 supplements using combined power of several studies on gestational length, reduction of PT labor, birth weight, or birth length. However, a meta-analysis which included four studies, three of which used long chain fatty acids, showed a significant decrease in early preterm birth (<34 weeks) and no decrease in late preterm birth [9]. An earlier meta-analysis of six studies demonstrated a nonsignificant increase in gestational length, birth weight, birth length, and risk for PT birth or low birth weight, but some question remains since the data from one study [5] was entered using the unadjusted increase in gestational length of 2.4 days not 6 days as published in the original report. The decrease in the incidence of preterm birth at <34 weeks in 350 women supplemented with 600 mg DHA/day from week 20 of gestation to delivery recently confirmed this effect [7]. A large Australian study which supplemented 2320 pregnant women with 800 mg DHA/day as fish oil from 20 weeks of gestation to delivery reaffirmed a decrease in risk for early preterm birth (adj OR .49, 95% CI, .56, 1.04) and found a significant (*p* < 0.05) 68 g increase in birth weight corresponding to a one-day increase in gestational length with the DHA supplement [14]. This increase in birth weight was similar to that seen in this study (46–56 g) with 300 and 600 mg DHA/day supplements, although the increase was not significant due to the smaller number of subjects.

Although some differences in DHA intake among Hispanics and non-Hispanic Whites and African American subjects were seen in this study population, intakes were similar to those previously reported. The 2007-2008 National Health and Nutrition Examinations Survey (NHANES) and What We Eat In America (WWEIA) show that women aged 20–29 years consume 0.05 grams (50 mg) DHA/day from food sources and women aged 30–39 years consume on average 0.06 grams (60 mg) DHA/day [15]. Judge used multiple 24-hour dietary recalls to measure baseline DHA intake of pregnant women in Connecticut and found that the average intake was 80 mg/day [16]. Chen et al. observed a range of 50–90 mg DHA/day intake among pregnant women with gestational diabetes mellitus living near New Jersey [17]. Among 20–40-year-old pregnant women from Canada, Friesen and Innis found that the mean DHA intake was 100 mg/day at 36 weeks of gestation [18]. Intakes are much lower than consensus recommendations for 200 mg DHA/day during pregnancy [19]. As previously reported, supplementation with DHA prevented the decrease in maternal DHA stores [11] and increased maternal RBC DHA at 36 weeks which we have previously correlated with decreased risk for preterm birth [3].

Overall, small to moderate fish consumption has a greater impact on preterm delivery than fish oil supplements. Studies of FO supplementation had greatest effects on women who eat little or no fish [7, 8] and tend to have no effect on gestational length in women with high baseline intakes of fish. FO or DHA supplements may not fully take the place of eating fish [12]. In the maternal-fetal network study, moderate fish intake decreased risk for reoccurrence of preterm birth, although the fish oil supplement did not. One reason may be that fish contain Vitamin D. Vitamin D deficiency has been associated with an increased risk for PT delivery [20]. Fish contain DHA, Vitamin D, and other nutrients which may be more effective than FO or DHA alone in reducing preterm delivery and promoting optimum neurocognitive development. For this reason, the 2010 Dietary Guidelines for Americans recommend that pregnant and breastfeeding women consume 8 to 12 ounces of fish per week [21].

### 4.1. Strengths and Limitations

The physiological significance of a 4-day increase in gestational length is unknown. However, a four-day increase may have a great impact on long term outcomes since recent data suggests birth between 37 and 38 completed weeks of gestation is associated with a 10% greater risk for attention-deficit hyperactivity disorder (ADHD) in school age children which increases to 30% in those born between 35 and 36 weeks of gestation [22]. Limitations of the study include the convenience sampling and nonblinded design of the nutrition education arm. This group, randomized only by clinic, may have impacted the result in that individuals that were more highly motivated by maternal nutrition may have volunteered to receive the education.

Another limitation was that baseline blood DHA levels and obstetric risk factors were not obtained in the nutrition education arm and these may have affected the outcome. However, there were no differences in initial RBC DHA levels or significant differences in demographics or obstetrical variables among any of the other three clinics serving the same population nor were there initial differences among treatment groups. Comparison of early preterm birth rates was only significant when the treatment groups were pooled. This result should be regarded with caution since the number of early preterm births was small and these were not preplanned comparisons. The strength of the study is that the effects of n-3 DHA from supplements and from food sources on gestational length were comparable in the same population group. Although the population studied was low income, the subjects were largely Hispanic, a group known to be at lower risk for preterm birth compared to other low income groups, so the results may not be generalizable to other populations.

## 5. Conclusions

Nutrition education or supplementation with DHA was shown to be effective in increasing gestational length in a low income population at relatively low risk for preterm birth. Improving DHA status through education or supplements is vital since maternal DHA levels are correlated with fetal DHA status and also represent enhanced DHA stores to support lactation and early infant growth and cognitive development. The nutrition intervention in this study would be expensive for clinics to implement but WIC programs routinely provide nutrition education at 3-month intervals to low risk pregnant participants and more frequently to high risk participants. Many WIC programs currently offer omega-3 DHA counseling and some even offer DHA-enriched foods. Alternative education methods may be explored, including existing programs such as Text4Baby which delivers gestational age appropriate health messages to pregnant and postnatal subscribers.

## Figures and Tables

**Table 1:** Subject characteristics by group and comparison of those who completed study and those who withdrew from study.

	Completed study *N* = 536	Withdrew from study *n* = 307 (*n*, %)
CON (*n*)	121	92 (43.2)
300 (*n*)	107	93 (44.9)
600 (*n*)	117	104 (47.0)
NEd (*n*)	191	18 (8.6)
RBC DHA at entry − % Total FA (mean ± sem)		
CON	3.43 ± 0.10	3.54 ± 0.12
300	3.67 ± 0.12	3.54 ± 0.12
600	3.47 ± 0.10	3.66 ± 0.11
Maternal BMI Kg/M^2^ (mean ± sem)		
CON	25.09 ± 0.50	25.69 ± 0.67
300	26.61 ± 0.55	25.06 ± 0.65
600	25.29 ± 0.54	25.31 ± 0.58
NEd	26.79 ± 0.46	26.24 ± 1.30
Maternal age years (mean ± sem)		
CON	25.1 ± 0.61	24.2 ± 0.48
300	24.5 ± 0.62	24.1 ± 0.45
600	24.3 ± 0.62	23.5 ± 0.48
NEd	27.0 ± 0.50	26.3 ± 1.32

CON: control; 300: 300 mg DHA/day; 600: 600 mg DHA/day; NEd: nutrition education.

**Table 2:** Race and ethnicity of subjects by group and completion status.

	Completed *n* = 563 (%)					Withdrew *n* = 289 (%)				
	CON	300	600	NEd	Total	CON	300	600	NEd	Total
White	7 (5.4)	8 (6.3)	9 (7.7)	29 (15.2)	53 (9.4)	8 (8.7)	6 (6.4)	8 (7.7)	5 (27.7)	27 (9.3)

African American	14 (10.8)	14 (11.0)	6 (5.1)	17 (8.9)	51 (9.0)	5 (5.4)	9 (9.7)	13 (12.5)	3 (16.7)	30 (10.3)

Hispanic	113 (87.6)	101 (79.5)	98 (83.7)	141 (73.8)	443 (78.5)	74 (80.4)	74 (79.5)	75 (72.1)	10 (55.5)	233 (80.6)

Native American	2 (1.5)	2 (1.6)	1 (0.8)	0 (0)	5 (0.9)	0 (0)	1 (1.1)	2 (1.9)	0 (0)	3 (1.0)

Asian	1 (0.8)	0 (0)	1 (0.8)	1 (0.5)	3 (0.5)	1 (1.1)	1 (1.1)	2 (1.9)	0 (0)	4 (1.4)

Other	2 (1.5)	2 (1.6)	2 (1.7)	2 (1.0)	8 (1.4)	4 (5.4)	2 (2.2)	4 (3.8)	0 (0)	10 (4.5)

CON: control; 300: 300 mg DHA/day; 600: 600 mg DHA/day; NEd: nutrition education.

**Table 3:** Fatty acid composition of DHASCO-S oil.

Fatty acid	Percent total
10:0	1.22
12:0	5.17
14:0	18.28
14:1	0.14
16:0	15.93
16:1	1.87
18:0	0.28
18:1n9	11.76
18:2n6	0.10
22:5n3	0.33
**22**:**6n3 (DHA)**	**44.83**

Source: Martek Biosciences.

All fatty acids present in concentrations >0.1% were reported.

**Table 4:** Birth weight and gestational age by intervention group^1^ (mean ± sem).

	Birth weight (g)	Birth length (cm)	Head circumference (cm)	Gestational age (days)	Postterm birth (*n*)	Induced labor >40 W *n* (%)	Induced labor >41 W *n* (%)
CON	3165.0 ± 44.96	49.41 ± 0.25	33.90 ± 0.20	271.6 ± 1.2^a,b^	1	8 (7.1)	6 (5.3)
300	3220.91 ± 47.78	49.92 ± 0.31	34.23 ± 0.21	275.0 ± 1.3	1	6 (5.4)	4 (4.5)
600	3210.56 ± 46.01	49.97 ± 0.30	33.99 ± 0.20	275.6 ± 1.3^a^	1	4 (3.4)	0
NEd	3218.89 ± 39.93	49.42 ± 0.25	33.77 ± 0.17	276.5 ± 1.1^b^	2	10 (5.2)	3 (1.6)

^1^Birth weight, birth length, head circumference, and gestational age were adjusted for ethnicity and maternal prepregnancy weight.

Values sharing superscripts are significantly different.

CON; control; 300: 300 mg DHA/day; 600: 600 mg DHA/day; NEd: nutrition education.

**Table 5:** Selected preterm birth risk factors in supplemented subjects in 129 control, 124 supplemented with 300 mg DHA, and 116 supplemented with 600 mg DHA (mean ± 1 sem).

	Parity	Previous preterm birth
CON	2.3 ± 1.3	0.07 ± .33
300	2.5 ± 1.6	0.09 ± .32
600	2.6 ± 1.5	0.10 ± .39
*p*	0.326	0.713

CON: control; 300: 300 mg DHA/day; 600: 600 mg DHA/day; NEd: nutrition education.

**Table 6:** Delivery events in supplemented subjects in 129 control, 124 supplemented with 300 mg DHA, and 116 supplemented with 600 mg DHA *n* (%).

	SROM	AROM	PROM	PPROM	Prolonged ROM	Preterm labor	Augmented labor	Induced labor
CON	52 (40)	53 (41)	4 (3)	8 (7)	4 (3)	18 (14)	43 (33)	16 (12)
300	51 (45)	62 (53)	3 (1)	3 (2)	1 (0.8)	10 (9)	36 (31)	7 (6)
600	41 (33)	75 (60)	1 (0.8)	4 (3)	3 (2)	12 (10)	33 (27)	12 (10)
*p*	0.168	0.514	0.426	0.304	0.473	0.357	0.500	0.226

CON: control; 300: 300 mg DHA/day; 600: 600 mg DHA/day; NEd: nutrition education; SROM: spontaneous rupture of membranes; AROOM: assisted rupture of membranes; PROM: premature rupture of membranes; PPROM: preterm premature rupture of membranes; ROM: rupture of membranes.

All NS different by Pearson Chi-Square analysis.
